# Pathological Anatomy

**Published:** 1838-04-01

**Authors:** 


### PATHOLOGICAL ANATOMY.

I. ILLUSTRATIONS OF THE ELEMENTARY FORMS OF DISEASE. By *Robert Carswell*, M.D. &c. &c. &c. Fasciculus Twelfth: INFLAMMATION.

II. LECTURES ON THE MORBID ANATOMY OF THE SEROUS AND MUCOUS MEMBRANES. In two Vols. By *Thomas Hodgkin*, M.D. &c. &c. &c. Vol. I. ON THE SEROUS MEMBRANES, &c.

### I.—INFLAMMATION.

THE Fasciculus before us concludes the Pathological Illustrations of Dr. Carswell. In a notice we are informed by that gentleman:—

"As originally announced, I have comprehended the Illustrations of the Elementary Forms of Disease in twelve fasciculi. There are, however, some subjects, interesting in themselves, and well adapted for illustration by means of coloured representations, viz. Calculi, Entozoa, and Monstrosities. These I propose to publish when a second edition of the work shall be required. They will form an appendix, and, along with any additions or alterations that may be made in the letter-press of the second edition, may be obtained by subscribers and others in possession of the first. I may observe that the order in which the fasciculi have been published is the reverse of that in which they should be arranged when bound. It was unavoidably adopted, and may be easily remedied on referring to the Table of Contents, which, for the reason assigned, must also serve as an index to the separate subjects."

This we think a judicious plan, and we trust that, at no distant period, the call for a second edition of the work will bring before the public further illustrations from the graphic pencil, and not unpractised pen of Dr. Carswell.

In the observations on Inflammation with which we are now favoured, our author treats successively of the Nature of Inflammation—of the Physical Characters of Inflammation, consisting in modifications of the natural colour and vascularity, the consistence, and bulk of parts—of the Diagnostic Characters of Inflammation—and, finally, of General Considerations on the Fluid Products of Inflammation.

It would be idle to insist on the necessity for an accurate acquaintance with the phenomena, the consequences, and the laws of inflammation. Dr. Carswell more particularly confines himself to the examination of the phenomena, and especially of those by means of which its existence may be determined after death.

### 1. *Nature of Inflammation.*

Our readers need not be told how many experiments have been performed, how many hypotheses have been framed, for the purpose of explaining the nature, or, as it is termed, the proximate cause of inflammation. Those experiments and theories have certainly not dispelled the obscurity that they were intended to remove, and there are few points on which the professional mind is less settled than on this particular subject.

Two leading hypotheses have for some time been before the public, and have numbered their several and eager supporters. The one hypothesis regards inflammation as essentially consisting in increased action of the capillaries—the other views it as depending on diminished action or debility. The same experiments have been appealed to by the advocates of opinions so opposite to one another, and although the party which deems inflammation to be debility of the capillaries is undoubtedly the predominating one, its opponent can adduce many plausible arguments, and can appeal to some staggering facts. Dr. Carswell steers a middle course, and endeavours to form a composite hypothesis out of the two that we have mentioned. His views cannot well be given more concisely, consistently with clearness, than he has given them himself. We shall therefore quote his words.

"I shall consider inflammation," he says, "as a disease the essential phenomena of which present themselves in two successive stages or periods, each of which is referable to opposite conditions of the physiological properties or functions of the affected part. Although these opposite conditions are sufficiently characterised by the changes which take place in the temperature of the inflamed part, and in the functions of circulation, secretion, and absorption, they are more especially so in those which occur in the sensibility and contractility. The changes induced in these two vital properties are, as I have already stated, the first that are observed in inflammation from the influence of stimuli, such as mechanical irritants. The first effect of mechanical irritation is an increase of the sensibility, soon amounting to pain, even in those tissues which, under ordinary circumstances, possess this property in a very low degree; and subsequently, or at the same moment, contraction, or an increased development of the contractile property, if not of the capillaries, of the arteries with which they immediately communicate. After a certain time, varying with the violence of the exciting cause, the sensibility diminishes or ceases to be manifested, for pain is no longer the consequence of the same cause. The contractility undergoes a similar change; the minute arteries cease to contract when stimulated, and they, as well as the capillaries and minute veins, are permanently dilated and distended with blood. That these two opposite states of the sensibility and contractility exist in inflammation, and occur in the order in which I have stated, might, independently of the evidence of the facts themselves, have been believed and understood; for they are but an exaggeration of the law of vital action, or of the physiological conditions of all parts endowed with these properties, a state of activity and repose, of increase and diminution, being the opposite and invariable consecutive conditions of both. It is hardly necessary to observe that these properties, both pathologically and physiologically considered, admit of these opposite conditions in so far only as the exciting cause acts in the strict sense of a stimulus; for if the agent to the influence of which they are subjected is of such a kind as to destroy, or, as is commonly said, to exhaust, instead of an increase there is at once produced a diminution, cessation, or extinction of both, and, consequently, of their visible signs. Such a state cannot, therefore, be regarded as a state of inflammation; for the first, or active, conditions of the disease have not existed. It is, however, a state which is frequently produced in experiments made to ascertain the effects of stimuli on the capillary circulation in inflammation, and then becomes an interesting illustration of the fact just stated; for although congestion takes place in the part thus debilitated, it is soon followed by the active phenomena of inflammation, or those which characterize its first stage, and a more rapid and extensive formation than usual of those of the second.

Next in succession and importance to these opposite states of the sensibility and contractility, are the changes which take place in the hydraulic conditions of the blood. In the first stage of inflammation, the circulation is accelerated, and a greater quantity of blood than natural passes into the capillaries; in the second stage, the circulation becomes impeded, and the blood which distends the capillaries ceases at last to circulate. That these changes in the circulation of the blood are the consequences of the difference in capacity of the capillaries in the first and second stages of inflammation cannot be satisfactorily ascertained; but what is of greater importance is the relation which exists between them and the primary lesions of the sensibility and contractility, as they correspond to the two opposite states of these properties, viz. an increased and diminished circulation, which implies opposite states of the agents which effect or regulate the transmission of the blood in the capillary vessels. There can be no doubt that other causes than these modify the circulation of the blood in the capillaries, in inflammation, independently of any increase or diminution of the propelling power. The diminished capacity of the smaller arteries in the first stage of the disease, and the dilatation which they present in the second, certainly offer, on hydraulic principles, a rational explanation of the increased and retarded circulation of the blood in each stage respectively. And increased vital affinity between the blood and the capillaries has also been supposed as a probable cause of accelerated circulation, and the subsequent accumulation of the blood, such as is observed to take place in erectile tissues during the state of turgescence; and changes in the blood itself, more especially its coagulation, which, in the second stage, must mechanically impede the circulation, and increase in degree and extent the local congestion. All these modifying causes of the capillary circulation must, however, be regarded as secondary in importance to those to the agency of which I have already ascribed the two essential changes which occur in it in the first and second stages of inflammation."

This is very well, but candour must admit that it leaves the difficulties nearly where it found them. It is difficult to reconcile the phenomena of active inflammation with the notion of debility, and impeded circulation in the capillary vessels. That has always constituted the *rational* difficulty. Every thing seems to shew that the circulation is more rapid—the artery leading to the inflamed part, say a finger, is felt to pulsate strongly, the colour of the inflamed is heightened and brightened—its temperature is heightened too—and surely all these circumstances ill-accord, or seem to ill-accord, with dilated capillaries *and* obstructed circulation in them. In the atonic form of inflammation, the theory is more at home, but the very fact of there being two forms of inflammation displaying such essential differences in their phenomena, is itself an apparent objection to any theory purporting to be an exclusive one. These difficulties, such as they are, serious or slight, are neither aggravated, nor, as it seems to us, much mitigated by Dr. Carswell's views. But we leave them without further comment to the consideration of our readers.

Dr. Carswell proceeds to observe that, during the first stage of inflammation, the vital properties of the blood undergo a manifest increase. A greater quantity of fibrine is formed, the plastic property of which is increased; for, besides its rapid organisation under favourable circumstances, it retains, when separated from the other constituents of the blood, its fluidity for a longer period, and contracts more firmly, than in the natural state. The unwonted vigour of the circulation in general, the increased temperature of the whole body, and the resistance opposed to the means employed in inflammation to weaken the vital powers, may also be regarded as connected with this state of the blood, or as inordinate effects of its vital agency through the medium of the nervous system. In this stage, too, the temperature is variously and greatly increased; the function of secretion is also for a time augmented; in glandular organs, however, only at the commencement; in serous tissues for a much longer period, and to a much greater degree. Absorption, if not increased, manifests its activity by the speedy effects of poisons locally employed in this stage.

During the second stage of inflammation, changes the reverse of those just described occur in rapid succession. The blood ceases to circulate, coagulates, and assumes a dark colour; the temperature sinks, and secretion, absorption, and nutrition are finally interrupted. If those conditions of the affected part are maintained for a certain time, new products are formed, or other diseased states are produced, as softening, suppuration, ulceration, and mortification, which are therefore denominated *terminations* of inflammation, and constitute separate subjects of investigation.

It appears to us that there is some confusion in Dr. Carswell's theory, or rather in this rider to it, for the theory itself we abstain from re-entering on. If we remember rightly, the advocates of the doctrine of debility appeal to experiments which prove, if they prove any thing, that such debility is a very *early* phenomenon. It could hardly indeed be otherwise, or it would not prove their case—that *it* is the proximate cause of inflammation. Dr. Carswell's remarks in the passage we have just borrowed, refer to quite an ulterior set of circumstances—the phenomena immediately preceding suppuration, or mortification. They do not then meet the question at issue, or obviate the difficulties which microscopic observations seem to oppose to analogy and reasoning. So, at least, it seems to us.

It seems to us, too, that to place suppuration and mortification in the same category, and to say that both ensue, when "the blood ceases to circulate, coagulates, and assumes a dark colour"—when "the temperature sinks, and *secretion*, *absorption*, and *nutrition* are finally interrupted," is to affirm what it would be difficult to prove, and what by no means gives us more definite ideas upon these subjects. But we waive the examination of these hypothetical points.

Dr. Carswell explains the signification of the terms adhesive, acute, gouty, syphilitic inflammation, and so forth—terms of practical consequence as representing modifications of the inflammatory phenomena, but, of course, not implying specific differences in the very essence of the inflammatory action itself. *That* is always the same.

2. *Physical Characters of Inflammation.*

Dr. Carswell examines those physical or anatomical characters of inflammation, which are peculiar to itself, excluding those of a physiological character, as well as those derived from the presence of coagulable lymph, pus, ulceration, and mortification. The physical characters in question consist in modifications in the natural colour and vascularity, in the consistence and bulk of the inflamed part.

*Redness and Vascularity.* These in an abnormal amount constitute the most conspicuous of the physical characters of inflammation.

"They vary much in degree, and are not equally present in all tissues. They occur also under several forms, which, as they indicate differences in the seat, mode of extension, stage or degree of inflammation, merit the special attention of the pathologist. They may be considered as constituting what may be called the *primary* and *secondary* forms of inflammation. The *primary forms* are as follow :—the *ramiform, capilliform, uniform, punctiform,* and *maculiform.* The first and second of these, as the terms imply, have their seat in the small arteries and veins, and in the capillaries respectively ; the third and fourth are produced by capillary injection, the uniform red colour which accompanies the former being the consequence of an accumulation of blood in the entire capillary system of a part ; the minute dotted or punctiform redness of the latter arising in the peculiar structure of the part, as in the villosities of mucous membranes, when the seat of inflammation, apart from the mucous tissue itself. The maculiform has also its seat in the capillary vessels, is sometimes produced by the accumulation of blood being greater in some points than others, but much more frequently by rupture of these vessels and extravasation, and hence may be called the *hæmorrhagic* form of inflammation. Exclusive of the influence of various modifying causes, but more especially of the quantity and quality of the blood, the several degrees of inflammation are expressed by each of these forms in the order in which they are enumerated, the ramiform indicating the least, the maculiform the greatest degree."

The following observations of Dr. Carswell's are general, and worthy of attention. Uniform redness, he writes, is best seen in dense vascular tissues, such as the skin, where it always occurs as the first physical sign of inflammation. Vascularity, on the contrary, is seldom conspicuous; and hence, when describing cutaneous eruptions, as the exanthemata, we employ the term redness rather than that of vascularity, to indicate the kind and degree of the inflammation which these eruptions present. Uniform redness is also observed in inflamed mucous membranes, but it is always preceded by the capilliform or punctiform injection. In these membranes also, the ramiform is remarkably conspicuous, and the maculiform or hæmorrhagic more frequent than in any other tissue. All these forms indeed, varying in degree, are met with combined in the mucous membranes, more especially of the digestive organs, the capilliform and punctiform being the first that appear, and the most characteristic of inflammation. In parenchymatous organs inflammatory redness is strongly marked; but from the complex structure of these organs, and the great quantity of blood with which they become impregnated during life or after death from a variety of causes, it is often extremely difficult to ascertain how far the redness depends on this circumstance or on capillary injection. In organs naturally of a pale colour, as the brain and nerves, lymphatic glands, testes, mammæ, &c., redness and the causes of it are sufficiently obvious. It depends on the uniform capillary injection of the first or second stage of inflammation, and is succeeded by the punctiform and hæmorrhagic, which indicate a progressive increase in the disease. In all the serous membranes uniform redness is produced in the same manner, the capilliform and punctiform injection appearing first, and giving rise, as the inflammation proceeds, to the formation of striæ or maculæ, which are distinguished from the former by their darker red colour. The ramiform injection never occurs in these membranes, this appearance, when it accompanies the other physical characters of inflammation, having its seat in the subjacent cellular tissue.

It has always appeared to Dr. Carswell, that even the capilliform injection of these membranes is in great measure produced by the penetration of the blood into their softened substance by the vis à tergo. He has never been able to discover blood-vessels in the arachnoid, in inflammation of the pia mater, except where it lies in contact with this membrane.

Dr. Carswell gives an useful hint or two. 1, The red colour which membranous tissues frequently present may be owing to the blood accumulated in the subjacent cellular tissue. 2. The amount of redness in tissues should be determined at once when they are exposed to the air. Under its influence the colour soon heightens, and becomes altogether fallacious.

"The *secondary forms* which inflammatory redness presents constitute an important feature among the other physical characters of inflammation, more especially in the cutaneous and mucous tisses. In the former they constitute the distinctive local characters of the exanthemata, and mark peculiarities in several of the papular, tubercular, vesicular, pustular, and scaly eruptions; in the latter they serve to distinguish the circumscribed and diffuse inflammations to which these membranes are subject, as well as the anatomical element in which they are seated, as the villous, mucous, and follicular structures. Thus in erythema, the most simple of the acute cutaneous affections, the secondary forms of redness occur in diffuse continuous patches of various extent, of a round, oval, or irregular form. The varieties which it presents are derived from the external appearances of the patches,—appearances which are produced by the degree of congestion of particular points, or of the whole of the inflamed portion of the cutis. When, for example, the redness is uniform, as in intertrigo, or when heat or a blister has been applied to the skin for a certain time, we have what is called erythema simplex. But when the cutis forms projections of various sizes, rendering the surface of the patches unequal or swollen, we have produced the varieties of this affection called erythema papulatum, tuberculatum, nodosum, &c."

It is not necessary to follow Dr. Carswell through his brief descriptions of the characteristic redness of erysipelas, measles, scarlatina, and so forth. They are necessarily too trivial to convey definite instruction.

The secondary forms of redness in inflamed mucous membranes are important from their indicating the precise seat of the inflammation. In the villous mucous membranes, as the digestive, the redness, at first punctiform and afterwards uniform, occurs in circumscribed patches; in the follicles it takes the form of these bodies, or when confined to their orifices, their basis, or both, appearing in dots, rings, or stellæ,—forms of inflammatory redness which distinguish it from every other. In the non-villous mucous membranes, or when confined to the mucous tissue, the redness occurs in diffuse patches which have no definite form.

Various shades of purple, brown, and black accompany, as is well known, the chronic or asthenic states of inflammation. They take place when the circulation has been long retarded, or when it has ceased in the capillaries, and especially when the inflammation has not terminated in suppuration or in effusion of coagulable lymph.

" When the circulation has only been retarded, the colour is brown or perhaps only of a high venous tint; and where there has been complete stagnation of the blood, the colour is black. The first degree of discolouration is seen in chronic inflammation of the eye, of the mucous membrane of the air-passages, and of the intestinal canal. In the eye it has almost always a ramiform aspect; in the intestinal canal it is more frequently punctiform, more particularly when seated in the villosities, producing a grey or slate colour, called by French pathologists *couleur ardoisée*. Sometimes, also, it occupies the orifices of the follicles, and appears in grey or dark-blue points; or having its seat in the mouths or basis of these bodies, in little rings or circles. In the mucous membrane of the air-passages it is much less common than in that of the stomach and intestines, is more uniform and much less marked; and in the mucous membrane of the urinary and generative organs it is often great in degree and considerable in extent. It is not frequently met with in serous membranes unless when complicated with other diseases; and in parenchymatous organs it is most conspicuous in the lungs, in which it exists alone or accompanies other diseases, as chronic hepatization and tubercles. It is important, however, to remember that similar discolourations are met with in tissues without being preceded by chronic inflammation, and which I have described under the head of Spurious Melanosis."

*Diminution of Consistence or Cohesion.*—This softening is a familiar consequence of inflammatory action. Commencing in the first stage, it may proceed to an extreme degree in the second. It seems chiefly to affect the cellular element of tissues or of organs.

In parenchymatous or spongy organs, and in all the tissues the structure of which favours the retention of the effused fluids, a state of solidification, called *hepatization* in the lungs, is produced; but although they then feel harder than natural when compressed, the diminution of cohesion which has taken place between their anatomical elements is rendered conspicuous by the facility with which they are penetrated, broken down, or crushed.

*Induration.*—It often accompanies chronic inflammation, and consists in an increased cohesion of the anatomical elements of the part affected. It is the result of the organization of the coagulable lymph, and is always attended with an increase of thickness or bulk, by the grey discolouration of chronic inflammation, and by a greater or less degree of opacity of all membranous tissues.

*Increase of bulk* always accompanies acute inflammation, in consequence of the greater afflux of fluids, sanguineous and serous, to the part, and of the effusion of coagulable lymph. Its effects may be highly important, from its obstructing apertures, and so forth.

### 3. *Diagnostic Characters of Inflammation.*

It is, of course, a great object, and often it is highly difficult, to distinguish inflammatory redness from that of other local accumulations of blood, produced during life or after death. A *mechanical imbibition* may occasion such local accumulations.

First, with respect to *local congestion, from a mechanical obstacle,* it is chiefly, if not exclusively, in mucous membranes, that a difficulty arises in distinguishing it from inflammation; and this difficulty is not felt unless the redness is general, and presents the ramiform character. Dr. Carswell points out very clearly the best mode of determining the question. Doubt, he observes, is removed by an examination of the neighbouring veins, which, in mechanical congestion, will be found dilated, tortuous, or even varicose, according to the degree and duration of the obstacle by which it has been caused. The congestion of the veins may likewise be traced to its cause—to a tumor compressing them, to disease of the liver, heart, or lungs, which has obstructed the return of the blood through them. In inflammation the local congestion commences in the capillaries, afterwards extends to the small veins, but never to large branches; in mechanical congestion the blood accumulates first in the trunks, which are always conspicuous, and afterwards in the branches and capillaries. It is only when mechanical congestion is combined with inflammation, that the anatomical diagnosis becomes difficult or impossible. In strangulated hernia and intus-susception, we are certain of the existence of both kinds of congestion of the affected portion of intestine from the nature of the causes in operation, viz. compression or stricture on the one hand, and the influence of a morbid stimulus on the other. But when mechanical congestion, produced by a remote cause, is combined with inflammation, it is impossible to detect the existence of this latter disease, or at least to distinguish the one from the other with any degree of certainty. The same may be said of the combination of these two kinds of local congestion, under similar circumstances, in other organs.

*Local Congestion from depending Position.* There is no certain mark of distinction between redness from this cause, and redness from inflammation occurring in the same parts. So we must generally experience some doubts when such parts are affected with respect to the exact nature of the affection.

But when local congestion exists in parts of organs which do not admit of the operation of this cause or of gravitation, and in the absence of a mechanical cause, we entertain no doubt of its being of an inflammatory nature, or the consequence of a morbid stimulus.

*Redness from Imbibition.*—There can be no question that this has been frequently confounded with inflammatory colouring, or, rather, mistaken for it. Dr. Carswell's directions for discriminating the one from the other are so precise and so satisfactory that we transcribe them. Whatever, he says, may be the circumstances which favour the red colour of imbibition, it never bears a near resemblance to that of inflammation. It is a mere dye of a uniform, almost scarlet red colour, generally limited to the lining membrane, without any other perceptible change of the coats of the vessel; whereas redness from inflammation is of a dull, rather pink tint, extending more or less to the other coats, accompanied by a fine capillary injection of the subjacent cellular tissue, and marked congestion of the vasa vasorum; the lining membrane is softened and opaque, or easily removed; the cohesion of the other tunics is diminished; they are also thickened or swollen, and infiltrated with serosity or coagulable lymph.

The following extract embodies the opinions of Dr. Carswell on another subject, and although we are not disposed to attach so much weight as he does to the circumstance he dilates on, yet we should be doing an injustice to our readers did we not lay his sentiments before them.

"I shall conclude this part of the subject by a few remarks on the *permanency* of the redness and vascularity of inflammation after death, as a character by which alone, and under doubtful circumstances, we may ascertain the existence of this disease, and distinguish it from the local congestions with which it may be confounded. And as the value of this character must, in part, be determined by ocular demonstration, it is only in cutaneous inflammations, and in those parts of the mucous membrane which are visible, that we can obtain positive evidence regarding it. The redness and vascularity of mechanical congestion, of position or gravitation, and of imbibition, differ, as we have seen, essentially from those of inflammation, in the mode of their production and other local circumstances. But they also differ essentially in other respects. Thus all these kinds of redness and vascularity are produced and maintained by appreciable causes which operate external to the vessels, without the blood, or the vessels themselves undergoing any change by which the vital properties of either are so modified as to render the redness or vascularity permanent after death. By means of ablution, pressure or scraping, both of these physical characters disappear in a short time. In inflammation it is far otherwise, the employment of the same means never removing, or effecting only a slight diminution of either. Injections too, which penetrate the capillaries in the former kinds of congestion, cannot be made to reach them in that produced by inflammation. It is necessary, however, to observe, that this state of the capillaries, and the redness which accompanies it, and which constitutes what I mean by the *permanency* of these physical characters of inflammation, are more or less decided by the degree or stage of the disease. In the highest degree or second stage, the permanency of both is most marked; in the first stage it is much less, the redness diminishing or disappearing entirely after death, in slight inflammations of the skin of short duration. This difference in the permanency of the redness and vascularity of inflammation in its two stages may be explained by the facts already noticed, viz. the absence of coagulation of the blood in the first and its occurrence in the second stage, in which also the fibrine unites with and penetrates the capillaries, thereby retaining the colouring matter of the blood, and producing occlusion of the vessels. Although, however, I have said that *redness* disappears after death in slight inflammation of the skin, some degree of increased *vascularity* remains, as is seen by comparing the diseased with a healthy part. And besides, it is of great importance to know that even in such slight cases of inflammation, the cutis undergoes changes which render the existence and extent of the disease very conspicuous after the disparition of the redness, and from one to two days after death. The affected parts only of the skin assume a purplish tint, and become infiltrated with bloody serosity, and the epidermis is detached from these parts much sooner than from those which were not affected by the inflammation. This *post-mortem* congestion, and a more rapid tendency to decomposition than in ordinary circumstances, are conditions which ought not to escape the notice of the pathologist, when it occurs in internal organs. They are sometimes the only morbid appearances which are met with in fatal cases of scarlatina, and especially in rubeola, and indicate the extent not only of the inflammation, but of the depressing influence which it must have exercised on the vital function of these organs."

4. *General Considerations on the Fluid Products of Inflamed Tissues.* These fluid products become valuable signs for the determination of the presence, degree, or stage of inflammation. Dr. Carswell despatches them briefly.

The first change observed in the process of secretion is an increase in the quantity of the natural fluid of the affected part. In proportion as the inflammation augments, the secretion diminishes, and it is suppressed in the second stage or most acute forms of the disease. During its progress, however, continues our able author, the qualities of the secreted fluids present important alterations. In inflammation of serous membranes, and at a very early period, the secreted fluid contains a quantity of albumen; afterwards, and as the inflammation increases, fibrine is added, and generally an admixture of the colouring matter of the blood, and lastly pus. The same order of succession is also observed to take place in the fluid products of inflamed mucous membranes. The mucous secretion, however, is, almost from the commencement of the inflammation, replaced by a serous fluid, which is often very abundant; this is succeeded by the presence of albumen and fibrine, and lastly of pus. The different degrees of fluidity, viscidity, and coagulability of the secretions generally of inflamed tissues are derived from the presence of the serum, albumen, and fibrine of the blood in various proportions. But the most important circumstances connected with these changes in the secretions of inflamed tissues, is the formation, or the separation from the blood, of two fluids of an opposite kind, viz. coagulable lymph and pus. The former, possessing vital properties, assumes, as is said, *spontaneously*, but no doubt in virtue of these properties, the solid form becomes organized, and fulfils the all-bountiful purposes which nature has assigned to it in the economy of life,—the reparation of those injuries so frequently the consequences of the disease of which it is the product. The latter, possessed of no plastic properties, being as it were the residue of the former and of the molecular structure of organs, by a disorganizing or destructive process, is essentially inert, and like all substances, incapable of being assimilated, is separated or removed from the body.

Dr. Carswell concludes with an observation regarding the products of inflamed mucous membranes, which is not unworthy of attention. Although sometimes, he says, assuming the form of membranous layers, cylinders, or tubes, they never become organized, never present the characters of the most simple of the analogous tissues, viz. cellular or serous tissue, so commonly met with in inflammation of serous membranes; and this is not owing to the influence of a physical cause, as their situation, but to a difference in their plastic properties. Under no circumstances has he ever met with a trace of blood-vessel in any of these forms of pseudo-membranous concretions. When the effusion which gives rise to them is accompanied by hæmorrhage, they are ocasionally reddened or present streaks of blood.

This terminates the observations of Dr. Carswell upon inflammation. As we observed before, this terminates, too, the work. With some faults, it has many and considerable merits, and Dr. Carswell has elevated himself to a high rank amongst the Pathologists of this country. His work will be consulted as always ingenious, generally valuable, and often highly philosophical. In the earlier fasciculi there was certainly a greater proneness to mere hypothesis than has appeared in the latter, and in subsequent editions we would recommend the author to modify the speculative portions. Pathological anatomy should be as exact as possible, and where conjecture is unavoidable, it should both be given and received as conjecture. On the whole, we can most conscientiously recommend all who have the means to purchase Dr. Carswell's work, and to patronize a production as well as an author of great merit.

The present Fasciculus contains four Plates as usual. As usual too, their execution is beautiful, and it would be hard to particularize any as more so than the rest. The first Plate exhibits Inflammation of the Cutaneous Tissue —the second, Inflammation of Mucous Membranes—the third, Inflammation of Serous Membranes—the fourth, Inflammation of Compound Tissues of Organs.

### II. MORBID ANATOMY OF THE SEROUS MEMBRANES.

In a former number of this Journal, we laid before our readers an account of the second lecture of Dr. Hodgkin, devoted to the examination of the morbid anatomy of the serous membranes in general. The third, the fourth, the fifth, and the sixth lectures, are occupied with the consideration of the lesions of the particular serous membranes—the arachnoid—the pericardium—the pleura—the peritoneum—the tunica vaginalis—and the bursæ.

We had hoped, that, ere this, the second volume of these lectures would have been given to the world, and that the morbid anatomy of the mucous membranes would have been added to that of the serous. But, unfortunately, since the publication of his first volume, the able and the amiable author has been attacked with sickness, and has been incapacitated from pursuing his useful, and honourable labours. We hope that the day is not remote, when his restoration to health and to science will afford delight, not only to his immediate friends, but to a whole profession.

We shall not go circumstantially into every particular connected with the lesions of the several serous membranes; many are familiar, and on those we shall not touch—some are not at all, or little so, and on them we shall insist at greater or less length.

### A. Lesions of the Arachnoid.

In treating of inflammation of the arachnoid, Dr. Hodgkin considers the membrane as consisting of four portions. Of these, the first comprises that which is external to the brain; the second, that which lines the ventricles; the third, the portion which belongs to the plexus choroides; and the fourth, that of the spinal cord.

*The Arachnoid external to the Brain.*—In most of the serous membranes, the inflammatory effusion is generally found within their close sacs. This is very rarely the case with the arachnoid. Villermé says that he has never seen it. But Dr. Hodgkin has, and in an instance not traumatic, where, of course, it is much more likely to happen. He details the case.

*Case.*—A young man, aged 23, had, for several months, been labouring under lumbar abscess. He never manifested any symptoms of affection of the head, until between four and five hours before the time of death. On inspection, besides other very interesting appearances, which it is needless here to describe, small but sufficiently evident and unequivocal recent adhesions were found between the arachnoid covering the dura mater, and that covering the pia mater. The far greater part of the effusion was, however, in the usual situation, beneath the arachnoid covering the pia mater: it was of a light yellow colour, and of a thin puriform consistence. It must not however be concealed, that, even in this case, the arachnitis appeared to depend on a local cause, which, though at a distance from the head, was nevertheless in a part most intimately connected with it. The lumbar abscess had formed a communication with the spinal canal, through the intervertebral foramina, by which the nerves pass out.

Another example, continues Dr. Hodgkin, of the product of the inflammation of the arachnoid occurring between its two polished surfaces is recorded by Rostan. In a patient who died in the Saltpêtrière, at the age of 64, who was originally rachitic, and who had twice been attacked with apoplexy, the last seizure of which took place eleven days before her death, a layer of puriform and albuminous matter, differing from half a line to a line in thickness, was found covering the surface of the arachnoid lining the dura mater in both of the temporal fossæ, and in the anterior and lateral part of the right hemisphere. It is expressly stated, that this layer had all the characters which belong to those found on the internal surface of the pleura.

But the cases related by Dr. Foville are very precise and satisfactory. He mentions six, the results of which are presented to us by Dr. Hodgkin.

Two considerable layers of false membrane extended over the whole of the internal surface of the arachnoid lining the dura mater and the external surface of that covering the brain, and were applied to these two surfaces; to which they were so slightly attached, as to admit of ready separation from them. They presented a considerable and uniform thickness, a good degree of firmness, and were of a yellow colour. There were but slight and partial adhesions between their opposed surfaces. There were thus four distinct membranous layers external to the brain: 1. The dura mater, with its serous lining; 2. The layer of false membrane corresponding to it; 3, A similar layer corresponding to the surface of the arachnoid investing the brain; and, 4. This portion of the arachnoid with the pia mater. The striking resemblance observable between all these cases induces Dr. Foville to believe that there is a special tendency to produce this form, whenever inflammation extensively affects the free surface of arachnoid. The distinctness and uniform diffusion of the two layers of false membrane he thinks attributable to the movements of the brain; and he points out the analogy to some cases of pericarditis, in which a similar disposition occurs. In all these cases of arachnitis, there was some effusion between the two layers of false membrane, In some, it was of moderate quantity, and in others more considerable. In the last case which the Doctor has met with, a considerable quantity of blood had been effused, which caused the patient's death. All these cases had commenced by an acute cerebral attack; which, in one instance, occurring in an old soldier, had been caused by a violent blow on the head. In the other instances he had not been able to ascertain the cause. There was a similarity in the symptoms presented before death in all these cases, in several of which they had existed for years. They exhibited a state of the most complete stupidity, accompanied by an apparent paralysis of nearly all the organs of sense. The patients actually resembled statues; with this difference, that when pushed, they walked; when set upright, they kept their balance; and when food was put into their mouths, they swallowed it. Their eyes, eye-brows, and other parts of the face, remained perfectly motionless. The sensibility of their skin appeared mechanical; for if a limb were pinched, they would withdraw it, but without any other evidence of suffering.

Films of false membrane have been repeatedly observed, by Dr. Guersent and Dr. Foville, in the temporal fossæ, and other parts of the base of the brain in children.

A puriform secretion in the arachnoid cavity is comparatively common as a consequence of injury. We need cite no instances of this sort.

Adhesions between the two layers of arachnoid are extremely rare. But they are met with, as the consequence of injury.

Morbid appearances resulting from inflammation are frequent enough on the attached surface of the arachnoid, or rather between it and the pia mater. The disturbance of the intellect produced by this form of arachnitis is familiar, and is explained by the fact, that the sub-arachnoid cellular tissue is traversed by the vessels of the cerebral structure, and by the conjecture, that the cortical substance of the brain is principally concerned in the manifestation of intellect.

Serum is most commonly found in the subarachnoid cellular tissue—sometimes lymph—more frequently a puriform fluid.

Dr. Hodgkin quotes from Dr. Foville, and we quote from Dr. Hodgkin, a description of the earlier appearances, characterising arachnitis.

"'The anatomical characters of meningitis are furnished,' as he observes, 'by the pia mater, by the arachnoid, and by the surface of the brain. The earliest stage is characterized by the delicate injection of the most minute vessels of the pia mater. The injection is so general, that it causes the external surfaces of the brain to appear of an extremely bright red, after a few minute's exposure to the air. The transparent serum naturally pervading the pia mater has become very scanty, and in some cases appears to be wholly wanting. The free surface of the arachnoid has neither the polish nor the moisture which it presents in its healthy state, but is slightly viscid, and sometimes dry and dull: the membranes are very readily torn, and are therefore detached in small fragments, which makes it tedious and difficult to separate them from the brain. The surface of the brain is manifestly reddened, minutely injected, and sometimes swollen in a remarkable manner. The redness is rendered most conspicuous when very thin slices are cut from the surface. There is sometimes such a deficiency of moisture in the brain, that the membranes appear to adhere to it; yet a little attention will convince us that this appearance is deceptive, and that it simply depends on the viscidity of the brain to which they are applied at those spots on which they are the most firmly pressed together. Such are the appearances observed in those rare cases in which death has quickly carried off the patient in the first period of acute meningitis.' " 75.

When serous effusion exists in the sub-arachnoid cellular tissue, the arachnoid itself is apt to become opaque. This opacity is considered by some as the result of maceration in the effusion—by others as the direct result of inflammation. Dr. Hodgkin inclines to the latter opinion, and believes that the appearance is one of the traces of an arachnitis of old date or chronic character. This is a matter difficult of determination. But of one thing we are certain. It is rarely that, in a person advanced in life, we do not find, on dissection, some serous effusion in the cellular tissue beneath the arachnoid, and some opacity of the latter membrane. If these, in a certain degree, are to be deemed evidence of old or of chronic arachnitis, then there are few aged persons who have not had the one or the other, without the occurrence of symptoms of either. We are inclined to place this alteration of parts in the same category as the alterations of the arterial coats, &c.—alterations which, though they resemble the consequences of inflammatory action, ought to be physiologically distinguished from them, and considered as natural degenerations and modifications of nutrition.

The appearance known by the name of glands of Pacchioni is another subject of doubt to pathologists. When the white substance on the arachnoid which produces it, exists in a larger quantity than usual, it is fair to suppose it morbid.

It has been disputed whether the internal surface of the dura mater is really lined by arachnoid. Rational hypothesis, analogy, and pathology seem to concur in inclining the balance towards the affirmative. Dr. Foville mentions, as the effects of recent inflammation between the arachnoid and dura mater, partial and irregularly circumscribed elevations of this part of the arachnoid, sensible both to the eye and to the finger, and accompanied by a brownish or a yellowish-red hue. When a portion of the dura mater and arachnoid exhibiting this state is detached from the calvaria, and pressed between the thumb and finger, as they are made to move upon each other the two membranes are found to separate readily in consequence of the altered state of the cellular membrane between them, which, in fact, is the structure chiefly affected. Bichat mentions a case of chronic inflammation of this part of the arachnoid, in which the membrane was sensibly thickened, and very easily detached from the dura mater.

Ossific deposits are often found between the arachnoid and the dura mater lining the cranium—more frequently upon the falx.

We do not perceive any thing to detain us in our outhor's observations on the *arachnoid of the ventricles*—or on the *arachnoid of the plexus choroides.* But we may cite a case of hæmorrhage between the dura mater and the arachnoid attached to its internal surface as additionally corroborative of the opinion that such a layer of arachnoid does exist. Supposing that no fallacy is mingled with the observation, it would indeed establish the opinion conclusively. Rostan has recorded three cases of a similar description.

*Case.* " On examining the body of a mulatto, about fifty years of age, who had lain for some time in a nearly comatose state, a considerable quantity of fluid escaped on opening the dura mater. It appeared to have been situated between this membrane and the arachnoid covering the pia mater. Blood was thinly but extensively extravasated over a great part of the inner surface of the dura mater. This extravasated blood was most copious on the left side, but by no means confined to it. It was in the greatest abundance about the base, but was likewise seen on the upper part. It presented, for the most part, a smooth polished surface towards the arachnoid covering the pia mater ; and was covered by a thin, even, firm, and generally transparent membrane, which in some parts, seemed to be double, having the effused blood between its layers. This delicate membrane could be nothing else than a portion of arachnoid reflected over the dura mater, and giving it its polished surface. Though very thin, it was too firm and resisting to be regarded as a false membrane formed from the fibrin of the effused blood. The dura mater, when this membrane was peeled off, lost its natural polish, and exhibited its fibrous texture. On different parts of the surface of the brain, but chiefly at the base, there were scattered irregular spots of an ochre-yellow colour, in which there was softening and loss of the substance of the brain ; but these appearances only extended to a very slight depth. I have met with another case of this kind, in which the blood extravasated between the arachnoid and dura mater was in part diffused, and in part collected into small defined portions, forming elevations of about the size of pepper-corns." 83.

*Spinal Arachnoid.*—From the cerebral, Dr. Hodgkin naturally passes to the spinal arachnoid. His observations on its lesions contain nothing which we need extract, with the exception of a few remarks upon its thickening.

As a consequence of old or chronic arachnitis, we sometimes find such a deposition of plastic lymph, either in the form of cellular membrane, or reduced to a denser substance, that the membrane appears thickened, and the distinction of its original layers is nearly or quite impossible. In this state, the arachnoid is not easily separated from the medulla, which is closely embraced, and even compressed by the contraction which has taken place. When these effects of arachnitis are found about the cervical portion of the cord, they are almost always combined with a similar condition of the arachnoid at the base of the brain, about the pons varolii and medulla oblongata. At the lower part of the cord it may exist separately.

" This thickening and contraction of the spinal arachnoid is well worthy of attention, in reference to practice. I believe it to be one of the causes of paraplegia, which must be more or less complete, according to the amount of compression exercised upon the chord ; and that, when the case is not extreme, progressive improvement may take place, both by the chord becoming accustomed to its pressure, and also by the slow and partial absorption of the adventitious deposit, which, though not wholly removed, may thus become of a laxer and more yielding texture. These considerations seem to explain the progressive but slow improvement which we sometimes observe in cases of paraplegia ; and should teach us to wait with patience, desisting from energetic measures, such as copious local depletion, deep and extensive setons, and other potent means of counter-irritation ; which may not only be resorted to in vain, but may increase the dangerous sloughing of parts exposed to pressure, while deprived of their ordinary degree of innervation. At the same time, I wish not to be understood as recommending that these cases, in their chronic state, should be left wholly to nature. I am persuaded that I have seen advantage derived from the careful and persevering use of means which are supposed to exert an influence upon the spinal chord; such as, nux vomica, carbonate of iron, and electricity, in conjunction with other means which the general health may require.

In those cases of arachnitis of the chord in which the inflammation had not yet passed into the chronic form, I have seen the vessels of the pia mater, at the affected part, considerably injected; and in one instance, the blood had acquired a blackish colour. Whether the arachnoid may have been injured by mechanical violence, or by exposure to cold, it is not uncommon to find the spinal chord itself more or less affected; being either softened, or having its cineritious matter unusually injected. Sometimes it presents a vermiform or granulated appearance; which may be attributed either to the compression of the contracted arachnoid, or to the turgescence of the affected part of the chord; or both causes may be combined." 88.

The cartilaginous or bony deposits occasionally found upon the cord, and sometimes regarded as the cause of chorea, tetanus, &c., are considered by Dr. Hodgkin as unequivocally morbid, and as indicative of such a want of integrity in the cord, as may predispose to grave affections of it.

### B. Lesions of the Pericardium.

So much has been said and written, of late, in reference to the inflammatory lesions of the pericardium, that we shall not attempt to follow our author into their details. We shall merely select a few passages for notice.

*a.* When there is a considerable admixture of inorganizable matter, the production of adhesions is prevented; in which case, the surface of the heart is not unfrequently covered with long, shaggy, soft, and very feebly organized villi. It is this state of the heart which has, in all probability, led to the idle and absurd stories of the heart having been found covered with hair. The heart of the great Messenian hero, Aristomenes, is said to have been hairy; and a similar account has been given respecting the heart of Old Parr, and of some other individual equally remarkable for his longevity.

*b.* Dr. Hodgkin alludes to the common and erroneous opinion, that the coagulable part of inflammatory effusion consists of albumen. Dr. Dowler has fully demonstrated, that, in the most plastic of these effusions, it is composed of fibrin.

*c.* "Acute pericarditis appears to be of by far the most frequent occurrence in young persons just arrived at, or but little past, the period of adolescence. It is probable that the danger of pericarditis is commonly much overrated, and that its comparative frequency is equally underrated. Louis's researches lead him to the conclusion, that pericarditis attacks, on the average, about one person in twenty." 96.

*d.* Ossification of the heart is a lesion more talked about than seen. The following are the results of Dr. Hodgkin's extensive opportunities of observation.

"Inflammation of the pericardium not unfrequently leads to a deposition behind the reflected, as well as on the close portion. Whilst on the free surface bony matter is of rare occurrence, and is seldom if ever found except in small isolated masses, on the attached surface the compact product of inflammation is apt to assume the form of bony plates. These cases, when the extent of ossification has been considerable, have been erroneously described as ossifications of the heart; and they have, I suspect, been somewhat exaggerated, by those who have wished to find in them an argument in favour of the arteries possessing the power of propelling the blood, and, consequently, muscular structure and function. One of the most considerable examples, with which I am acquainted, is seen in a preparation belonging to our Museum. The osseous plate occupies a large portion of the base of the heart, where it forms a complete bony ring. The apex of the heart is, however, left at liberty; and, consequently, the contractions of the ventricles, though doubtless much interfered with, were by no means wholly prevented. It was taken from a young woman twenty-two years of age, who had laboured under ascites for ten months, and had been tapped ten times. The ascites was preceded by an attack of dyspnœa with orthopnœa, which subsided in a fortnight. No symptom connected with the thoracic viscera was afterwards noticed." 97.

*e.* We shall quote Dr. Hodgkin's hypothesis of the formation of those opaque white patches, which are occasionally seen on the surface of the heart.

"It is very common to find one or more opaque white patches on the surface of the heart. Dr. Baillie says that they may be dissected off; and Laennec says that this is frequently the case. I have met with one or two instances in which this might have been done; but I certainly agree with Corvisart, in thinking that, in by far the greater number of cases, these patches depend on a deposit on the attached surface. They are generally found on the anterior part of the right ventricle, and rather nearer to the apex than to the base of the heart. They are, however, by no means confined to this situation. Respecting the cause of these spots, which can scarcely be regarded as a morbid appearance, nothing certain is known. From the circumstance of their being often found immediately under the sternum, and from their being occasionally met with on other parts of the heart to which a firm and resisting body has been unusually opposed—as, for example, where a bony deposit has taken place beneath the reflected pericardium, or when an uneven and remarkably indurated liver has, even through the diaphragm, presented an unequal pressure against a particular part of the heart—I have thought it probable that such pressure, aided by the movements of the heart itself, may have led to the production of these spots. These formations may certainly take place at a very early period of life. I have met with one rather loose and thick, but in other respects perfectly resembling those found in the adult, on the right ventricle of the heart of a child only ten weeks old. Similar thickening of the close pericardium sometimes marks the course of the coronary arteries and their branches; and this circumstance, amongst others, tends to confirm the idea which I entertain, as to its mode of formation." 98.

We cannot say that Dr. Hodgkin's theory appears to us very plausible. We have seen the patches so close upon the auriculo-ventricular groove, as to render it highly improbable that they could have been produced by any pressure on the sternum.

### c. LESIONS OF THE PLEURA.

This lecture derives its main interest, indeed its bulk, from its offering a very complete account of the important affection—empyema. This account is too lengthened for our present purpose, and we shall quit, for the moment, Dr. Hodgkin's work, and the subject of Morbid Anatomy.

